# Observations on Tubercular Hip Joint Diseases

**Published:** 1910-01

**Authors:** L. Sexton

**Affiliations:** New Orleans, La.


For Texas Medical Journal.

### Observations on Tubercular Hip Joint Diseases.

BY L. SEXTON, M. D., NEW ORLEANS, LA.

Tubercular hip joint disease is a very common affliction among poorly housed and badly nourished children of cur cities. This trouble has been prevalent for many years under various names of white swelling, morbus coxarius, coxitis and hip joint disease.

Tuberculous disease of the hip joint has been confounded with other inflammations of the joint and bones, such as osteomyelitis, rheumatism, gout and arthritis deformans, but is easily differentiated by the tuberculin and other tests.

### MODE OF INFECTION.

It may have its starting point from an injury, or it may come on spontaneously, but in either event infection with the bacillus of tuberculosis is always present.

It is supposed to gain entrance into the joint through the lymphatics from drinking tuberculous cow's milk, and experiments prove that the bacillus may also be admitted through the blood current. That there are other sources of infection, as tuberculous food, is very probable.

Tuberculous hip joint disease has a predilection for strumous children, but at the same time we often find robust and healthy children beginning to limp without traumatism or rheumatism, finally developing all the symptoms of the tuberculous hip.

The disease may assume the form of osteo-arthritis or synovitis.

If it happens to be in the knee joint, where it is superficial and easily examined, you can make out the distinction between the two much more easily than when the hip joint with its large covering of muscles is involved.

### PROGNOSIS.

Billroth said many years ago that 27 per cent of tuberculous hip joint case died within sixteen years under the old plan of management. Koenig says that 16 per cent out of 117 cases operated on for joint tuberculosis died within four years after the operation. He found that 60 per cent of these cases have secondary lesions and that 56 per cent of the cases dying of Pott's disease had tubercular deposits in other organs of the body. Under present management results have been very much better.

PROMINENT SYMPTOMS.

Symptoms of tuberculous hip are pain often referred to knee joint, deformity, perishing of the muscles of the thigh, anterior lumbar curvature, upward tendency of the pelvis on the diseased side, and adduction with shortening, as the disease progresses.

The limb of the affected side is usually flexed, and may be ab- or adducted in varying degrees. When the patient is lying down on flat table the spine is arched anteriorly. In each case the loss of range of motion of the hip on the affected side is one of the first symptoms noticed.

Flexion of the leg is a common diagnostic symptom of tubercular hip disease. Muscular wasting from non-use causes the buttocks to flatten and perish. Atrophy of limb on account of hip joint disease comes from growth cessation and non-use. Shortening of the limb may be produced both by cessation of growth and by absorption of the head of femur acetabulum and pathological dislocation of the hip joint usually backwards and upwards.

The cancellous ends of bones are the ones most often attacked by the tubercle bacilli. The epiphyses, the medullary canal or shaft just under the periostium may be the beginning point of any tuberculous joint. The tubercular process may for man abscess in the neck or body of the bone; in fact, the disease may involve any portion of the joint, the synovial membrane and ligaments may also become eroded and pulpy.

The acetabulum is likewise affected by being increased in size and depth. The irritation of the diseased process brings about a tonic contraction of the muscles, keeping the limb in a flexed position, usually inversion or eversion, as the case may be.

Owing to the fact that the knee is supplied by the anterior crural, sciatic and obturator nerves, the pain is as often located in the knee joint as it is in the hip; hence all knee joint pains cause us to think of hip joint disease at once.

At first the thigh seems to be wasting, the leg may apparently be lengthened. The pelvis is tilted upward on the affected side, there is often spinal lordosis (anterior lumbar curvature), with lateral curvature higher up, the convexity being towards the diseased side.

Early rigidity of the hip joint is noted; any attempt to flex the limb on the abdomen raises the pelvis from the examining table.

Nature effects its cure in hip joint disease by fixation, adduction and flexion. From this fact it is natural to infer that if the joint is put absolutely at rest without this flexion and adduction it ought in the nature of things to get well without this adduction or deformity.

### TREATMENT.

When Dr. Lorenze was in this country, the object of treatment in his cases was to get the joint well with ankylosis, which could later be brought into at least partial use.

Now, following the teachings of this great specialist, it would seem advisable to fix the joint just as soon as possible after the discovery of the disease.

There are many appliances for fixation, traction and protection accomplished by different methods.

Any apparatus intended for the cure of hip joint disease has to be designed with two objects in view, namely, the prevention of adduction and flexion of the diseased limb. This mechanical proposition is met in some cases by applying long zinc oxide adhesive straps to the limb (it previously having been shaved) from the middle portion of the thigh down around the heel, returned on the opposite side of the thigh to the point of beginning. A small block with hole for rope is placed just under the heel wide enough to prevent these adhesive straps from pulling together so as to cramp the ankle.

If the mattress is hard the adduction can be partially overcome by placing under the limb a wide piece of plank upon which is placed on the inner side of the limb a long sand bag extended from the perineum to the ankle. This adduction can also be obtained to a certain extent by the application of the zinc oxide plaster in such a way as to make the traction greater on the inner side of the limb, having the patient's body so placed on the bed that when this traction is made it will then evert the limb.

The matter of fixation of joint surfaces may be obtained by a plaster cast which extends from the knee on the diseased side up to the ensiform cartilage, making a stiff dressing with the hip joint entirely surrounded in one continuous splint. This affords ample protection and fixation. As a matter of course any apparatus of this kind should be supplemented with crutches and elevated shoe when the patient tries to walk.

The zinc oxide plaster extension apparatus can be applied to the limb before this plaster cast is put on, so that the weights and extension can be used as soon as the patient retires.

To recapitulate: The plaster of Paris band meets the indication for fixation, the zinc oxide straps traction, and the elevation of the shoe sole on the opposite foot and crutches meet the ambulatory indication for treatment.

There is difference of opinion as to whether a moderate amount of motion in tuberculous hip joint is not better than absolute fixation.

In a rather limited experience it would seem to us that the less movement of two diseased surfaces together the better for the part involved.

Some criticism has been made to the plaster Paris cast, that it kept the limb so still, it encouraged disuse, thereby increasing the atrophy of the part. Granting this to be the truth, which certainly is not always the case, it would perhaps not take a treatment of over six months to relieve or greatly ameliorate the condition. Now, whatever is lost in growth in this short period of time can be readily overcome by massage, gradual Swedish movements, vibratory and other expedients for developing muscular activity. This muscular atrophy dwindles into insignificance compared to the tubercular processes which is the main desideratum for correction. Traction when applied to a sound limb increases its length very materially and when applied to a diseased limb, not only prevents shortening to any great extent, but keeps the two diseased surfaces pulled apart.

Under the above outlined plan we have had three cases brought to a successful termination with a moderate degree of motion in two and ankylosis in one.

There is nothing original to be claimed from the plan outlined here, unless, perhaps, it is the method of roller and extension that makes the upright to which the pulleys are attached applicable to almost any bed which can be held in place by a couple of ordinary one-inch screws. The fixation of the hip is obtained by the plaster of Paris; the out of door method of treatment is obtained by a couch or roller chair on the gallery (weather permitting); the extension by weights or pulleys can be utilized half or all the time as the case may require.

Up to 1875 the long Liston splint used in fractures was depended upon. Since that time fixation for the body and same form of extension have taken the place of the Liston splint.

It is with this treatment by extension and counter-extension that this paper has to deal. I have often found it impossible to adopt the Levis apparatus to the bed used by the majority of families, as all cases can not be treated at the institution with the metal beds and attachments. I have devised the upright piece of steel which is shown here, and which can be screwed to any bed with foot board by running the extension rope under one pulley and over two, any weight which can be attached and suspended free of friction with the bed.

The foot of the sound side can be depended upon for the counter-extension or keeping the weight from pulling the patient too far down in bed. It may be necessary at times to pass a wide band padded over the perineum and to attach it to the head of the bed.

The amount of weights to be used depends upon the tension of the muscles to be overcome. The weights should be gradually increased until the shortening is reduced. Under this treatment the curvature of the spine corrects itself about as soon as the deformity of the limb.

This weight is worn all night and a greater part of the day, but the patient is usually allowed to move around into the open air and to move about in rolling chair while keeping the hip as still as possible.

This is often done by a spica bandage around the hip.

There is very often a curvature of the spine or tubercular process in the vertebral column in those cases of tubercular hip trouble.

It is just as important to extend or pull these diseased vertebræ apart as it is to extend or to fix the hip joint.

This weight and pulley, though applied to the leg primarily, is just as efficient in extending and resting the spinal column.

### RECURRENCE.

Because tuberculosis in the hip joint may be arrested and all evidence of the disease except ankylosis and atrophy seems to disappear, it is important to keep up the forced feeding, fresh air and rest for at least six months after the last symptom of the disease has disappeared.

The Thomas splint was originally used for this period of the treatment with the shoe on the well side raised two inches in order to permit the patient to get about on crutches, leaving the diseased limb in a fixed position. Instead of the Thomas splint a spica plaster of Paris bandage may be applied, which permits more freedom of motion on the part of the patient, but keeps the hip joint at rest just the same. Again, after the hip joint seems perfectly well, there may be a metastasis in which tuberculous process may set up in some other organ of the body.

As a matter of course forced feeding and the best hygienic surroundings are just as essential in the treatment of joint tuberculosis as it would be in that affecting the lungs.

